# The Infraoptic or Infrachiasmatic Course of the Anterior Cerebral Artery Emerging an Elongated Internal Carotid Artery

**DOI:** 10.3390/tomography8050188

**Published:** 2022-09-06

**Authors:** Dragoş Ionuţ Mincă, Mugurel Constantin Rusu, Petrinel Mugurel Rădoi, Alexandra Diana Vrapciu, Sorin Hostiuc, Corneliu Toader

**Affiliations:** 1 Division of Anatomy, Department 1, Faculty of Dental Medicine, “Carol Davila” University of Medicine and Pharmacy, RO-020021 Bucharest, Romania; 2 Division of Neurosurgery, Department 6—Clinical Neurosciences, Faculty of Medicine, “Carol Davila” University of Medicine and Pharmacy, RO-020021 Bucharest, Romania; 3 Clinic of Neurosurgery, “Dr. Bagdasar-Arseni” Emergency Clinical Hospital, RO-041915 Bucharest, Romania; 4 Department of Legal Medicine and Bioethics, Faculty of Dental Medicine, “Carol Davila” University of Medicine and Pharmacy, RO-020021 Bucharest, Romania

**Keywords:** internal carotid artery, anterior cerebral artery, optic nerve, optic chiasm, neurosurgery, circle of Willis, skull base, brain, clinoid process, carotid dolichoectasia, third ventricle, pituitary gland

## Abstract

(1) Background: The normal A1 segment of the anterior cerebral artery (ACA) has a supraoptic course. The proximal infraoptic course of an A1 segment leaving the internal carotid artery (ICA) near the origin of the ophthalmic artery is a rare possibility. This study aimed to determine the prevalence and detailed anatomy of infraoptic A1 segments. (2) Methods: We retrospectively studied 145 computed tomography angiograms from 92 male and 53 female cases, with ages varying from 61 to 78 y.o. (3) Results: In 21/145 cases, infraoptic or infrachiasmatic horizontal-medial courses of A1 segments that emerged distally from the ICA were found. Distal infraoptic A1 segments were bilateral in 16/145 cases and unilateral in 3/145 cases. Infrachiasmatic A1 segments were found bilaterally in 2/145 male cases. All the infraoptic/infrachiasmatic A1 segments left long ICAs with low bifurcations. In 7/34 sides with distal infraoptic or infrachiasmatic A1 segments, supracarotid courses were present. In one female, the right A1 segment had an anterior supraclinoid, supracarotid and infraoptic course. In two female cases with a bilateral distal infraoptic A1, the segment was almost contacting the respective posterior cerebral artery. (4) Conclusions: In cases with dolicho(ectatic) ICAs, the A1 segments could have infraoptic and supracarotid courses the neurosurgeons should be aware of.

## 1. Introduction

According to its course, the internal carotid artery (ICA) is described as having seven segments: C1 (cervical), C2 (petrous), C3 (lacerum), C4 (cavernous), C5 (clinoid), C6 (ophthalmic) and C7 (terminal and communicating) [1]. The C4 ICA segment exits the cavernous sinus through the proximal dural ring in its superior wall [1]. The short C5 ICA segment begins at the proximal dural ring and ends at the distal dural ring, where the ICA enters the subarachnoid space [1]. The C6 ICA segment arches posterosuperiorly, being the most proximal intradural portion of the supraclinoid ICA [1]. The last C7 ICA segment is the segment between the origin of the posterior communicating artery (PComA) and the carotid bifurcation into the anterior (ACA) and middle (MCA) cerebral arteries [1].

Normally, the ICA terminates inferior to the anterior perforated substance, bifurcating into the ACA and MCA [1,2]. Of the two terminal branches of the ICA, the ACA is smaller [2]. The left and right ACAs are united by the anterior communicating artery (AComA). At the base of the brain, the ICA system builds the circle of Willis (cW) with the posterior cerebral arteries (PCAs) branching from the basilar artery (BA). The PComA unites the ICA and the PCA. The arterial circle of Willis is one of the most famous eponymous structures in human anatomy [3]. Anatomic variations in the circle of Willis are the rule, not the exception [1].

Commonly, the precommunicating A1 segment of the ACA courses above the ipsilateral optic nerve to move in front of the optic chiasm. The ACAs continue above the AComA with the postcommunicating A2 segments.

Infraoptic A1 segments of the ACA were found to originate from a low bifurcation of the ICA (a bifurcation of the proximal intradural segment of the ICA); such infraoptic A1 segments have a horizontal–medial course below the ipsilateral optic nerve, and further turn superiorly on the anterior side of the optic chiasm [4,5,6,7]. Such infraoptic A1 segments could even pierce the ipsilateral optic tract [8]. Infraoptic A1 ACAs are rare findings.

We, therefore, aimed to use computed tomography angiograms to document the incidence of infraoptic A1 segments. We serendipitously found a different pattern of such topographic variants: infraoptic A1 segments resulted from distal bifurcations of the intradural ICA.

## 2. Materials and Methods

We conducted a retrospective study on 145 computed tomography angiograms (CTAs) to evaluate the occurrence of infraoptic ACAs. Cases were selected from regular CTA studies performed for suspected cerebrovascular disease. Inclusion criteria were the age of the subjects (>18 years), adequate quality of the angiograms and no pathologic processes distorting the anatomy of the anterior circle of Willis. Exclusion criteria were pathological processes distorting the arterial anatomy and degraded or incomplete computed tomography scans. There were 92 male and 53 female cases, with ages ranging from 61 to 78 y.o.

All subjects gave their informed consent for inclusion before they participated in the study. The research was conducted following principles from The Code of Ethics of the World Medical Association (Declaration of Helsinki). The responsible authorities (affiliation 3) approved the study (approval no.2093/1 March 2022).

The CTAs were performed with a 32-slice scanner (Siemens Multislice Perspective Scanner, Erlangen, Germany), with a 0.6 mm collimation and a reconstruction of 0.75 mm thickness, with 50% overlap for a multiplanar maximum intensity projection and three-dimensional volume rendering technique, as described previously [9]. The cases were documented using Horos for iOS (Horos Project) [10]. The course of the ACA in relation to the optic nerve was verified on coronal and sagittal reconstructions.

The cases were documented bilaterally for the different topographic patterns of the A1 segment course, referred to as the optic nerve: type 1—supraoptic course of the A1 segment or absent A1 segment; type 2—infraoptic course of the A1 segment; type 3—infrachiasmatic course of the A1 segment beneath the optic chiasm.

## 3. Results

**Figure 1:**
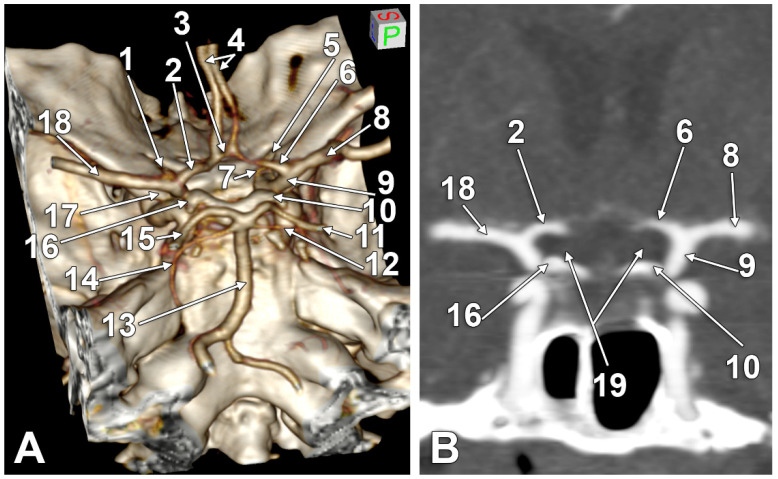
(**A**) Three-dimensional volume rendering of the skull base, left posterosuperolateral view. (**B**) Coronal cut through the internal carotid arteries bifurcation. Supraoptic A1 segments of the anterior cerebral arteries. 1. Left optic canal; 2. supraoptic A1 segment of the left anterior cerebral artery; 3. anterior communicating artery; 4. A2 (postcommunicating) segments of the anterior cerebral arteries; 5. right optic canal; 6. supraoptic A1 segment of the right anterior cerebral artery; 7. right ophthalmic artery; 8. right middle cerebral artery; 9. right internal carotid artery; 10. right posterior clinoid process; 11. right posterior cerebral artery; 12. right superior cerebellar artery; 13. basilar artery; 14. left superior cerebellar artery; 15. left posterior cerebral artery; 16. left posterior clinoid process; 17. left anterior clinoid process; 18. left middle cerebral artery; 19. optic nerves.

In 124/145 cases (85.52%), type one supraoptic courses of the A1 segments of the ACAs (Figure 1) were found. On three-dimensional renderings, the origin of a supraoptic A1 segment was observed above the horizontal levels of the optic canals and clinoid processes.

**Figure 2:**
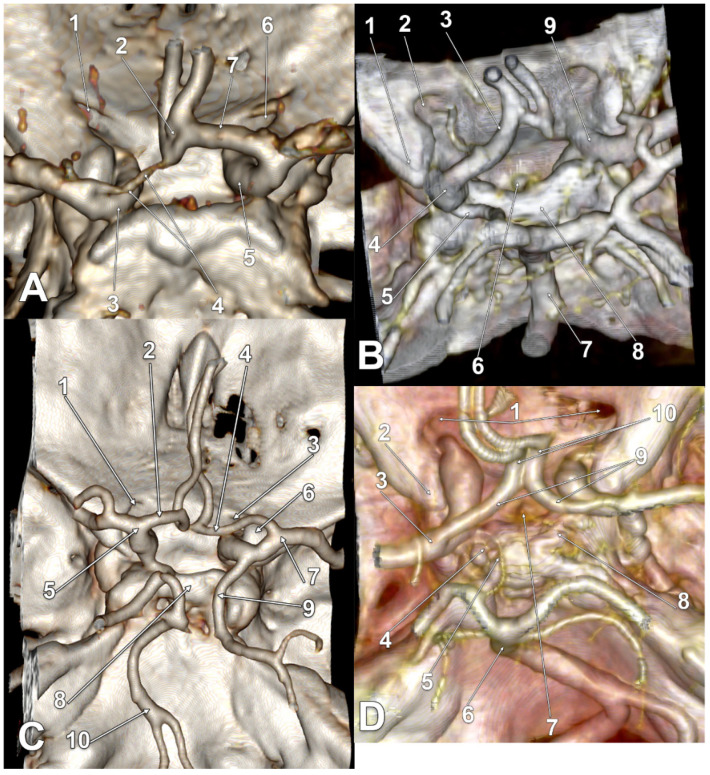
(**A**) Three-dimensional volume rendering of the skull base, posterosuperior view. Infraoptic, supracarotid and hypoplastic left anterior cerebral artery. 1. Left optic canal; 2. fenestrated anterior communicating artery; 3. left internal carotid artery; 4. A1 segment of the left anterior cerebral artery; 5. right internal carotid artery; 6. right optic canal; 7. A1 segment of the right anterior cerebral artery. (**B**) Three-dimensional volume rendering of the skull base, posterosuperior view. Bihemispheric infraoptic left anterior cerebral artery. 1. Left anterior clinoid process; 2. left optic canal; 3. bihemispheric left anterior cerebral artery; 4. left internal carotid artery bifurcation; 5. posterior communicating artery; 6. pituitary stalk; 7. basilar artery; 8. dorsum sellae; 9. right internal carotid artery. (**C**) Three-dimensional volume rendering of the skull base, posterosuperior view. Supraclinoid, infraoptic and supracarotid right anterior cerebral artery. 1. Left optic canal; 2. supraoptic A1 segment of the left anterior cerebral artery; 3. right optic canal; 4. infraoptic A1 segment of the right anterior cerebral artery; 5. left internal carotid artery bifurcation; 6. right anterior clinoid process; 7. right internal carotid artery bifurcation; 8. dorsum sellae; 9. fetal type of posterior cerebral artery; 10. basilar artery. (**D**) Three-dimensional volume rendering of the skull base, posterosuperior view. Posterolaterally displaced carotid bifurcations and resulting infrachiasmatic A1 segments of the anterior cerebral arteries. 1. Optic canals; 2. left anterior clinoid process; 3. left internal carotid artery bifurcation; 4. left posterior clinoid process; 5. left posterior communicating artery; 6. basilar artery; 7. pituitary stalk; 8. right posterior clinoid process; 9. A1 segments of the anterior cerebral arteries; 10. prechiasmatic loops of the A2 segments of the anterior cerebral arteries.

**Figure 3:**
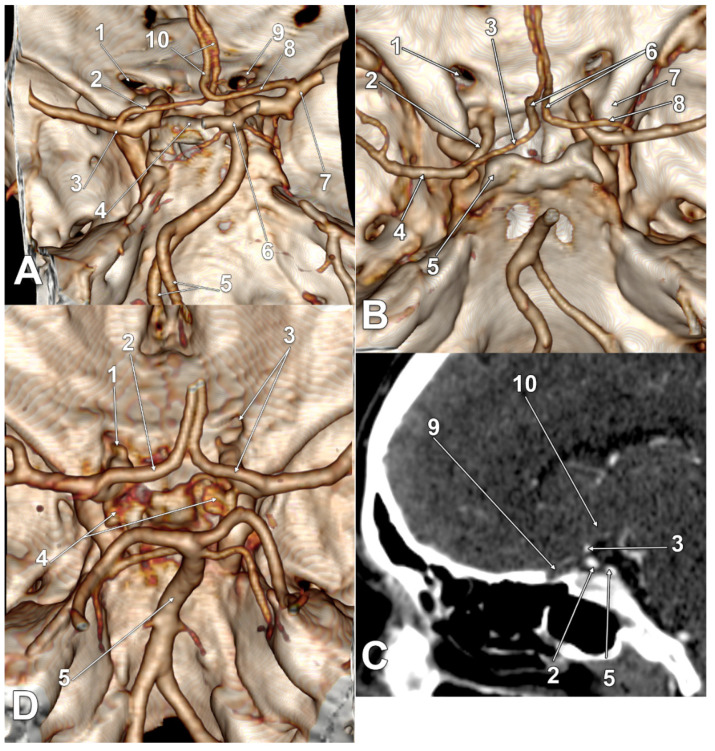
(**A**) Three-dimensional volume rendering of the skull base, right posterosuperior view. Bilateral supracarotid and infrachiasmatic anterior cerebral arteries. 1. Left optic canal; 2. A1 segment of the left anterior cerebral artery; 3. left internal carotid artery bifurcation; 4. dorsum sellae; 5. twisted vertebral arteries; 6. basilar artery bifurcation; 7. right internal carotid artery bifurcation; 8. A1 segment of the right anterior cerebral artery; 9. right optic canal; 10. twisted A2 segments of the anterior cerebral arteries. (**B**) Three-dimensional volume rendering of the skull base, posterosuperior view. (**C**) Sagittal slice through the right optic nerve of the case in (**B**). Posteriorly displaced carotid bifurcations and resulting infraoptic A1 segments of the anterior cerebral arteries. 1. Left optic nerve canal; 2. left internal carotid artery; 3. infraoptic A1 segment of the left anterior cerebral artery; 4. left middle cerebral artery; 5. left posterior clinoid process; 6. prechiasmatic A2 segments of the anterior cerebral arteries; 7. right anterior clinoid process; 8. infraoptic A1 segment of the right anterior cerebral artery; 9. left optic nerve; 10. optic chiasm. (**D**) Three-dimensional volume rendering of the skull base, posterosuperior view. Posteriorly displaced carotid bifurcations and resulting infraoptic A1 segments of the anterior cerebral arteries. 1. Left ophthalmic artery; 2. A1 segment of the left anterior cerebral artery; 3. right optic canal, A1 segment of the right anterior cerebral artery; 4. posterior clinoid processes; 5. basilar artery.

**Figure 4:**
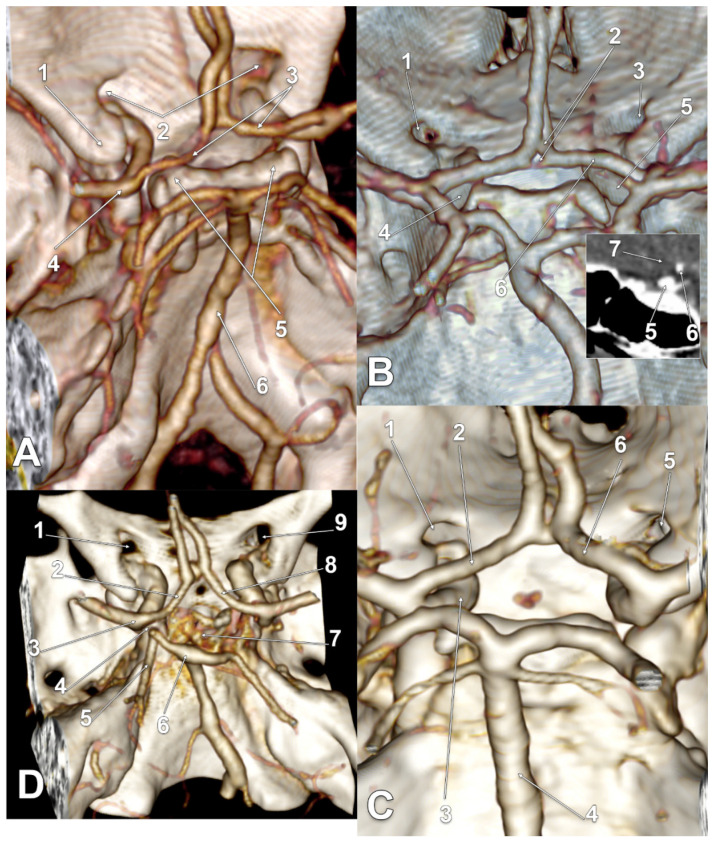
(**A**) Three-dimensional volume rendering of the skull base, left posterosuperior view. Bilateral infraoptic anterior cerebral arteries. 1. Left anterior clinoid process; 2. optic canals; 3. A1 segments of the anterior cerebral arteries; 4. left internal carotid artery bifurcation; 5. posterior clinoid processes; 6. basilar artery. (**B**) Three-dimensional volume rendering of the skull base, posterosuperior view. Inset: sagittal slice through the right optic nerve. Bilateral infraoptic anterior cerebral arteries. 1. Left optic canal; 2. duplicated anterior communicating artery; 3. right optic canal; 4. left internal carotid artery; 5. right internal carotid artery; 6. infraoptic A1 segment of the right anterior cerebral artery; 7. right optic nerve. (**C**) Three-dimensional volume rendering of the skull base, posterosuperior view. Bilateral infraoptic anterior cerebral arteries; supracarotid left A1. 1. Left optic canal; 2. A1 segment of the left anterior cerebral artery; 3. left internal carotid artery; 4. basilar artery; 5. right optic canal; 6. A1 segment of the right anterior cerebral artery. (**D**) Three-dimensional volume rendering of the skull base, posterosuperior view. Bilateral infraoptic anterior cerebral arteries. Rotated basilar artery bifurcation. 1. Left optic canal; 2. A1 segment of the left anterior cerebral artery; 3. left internal carotid artery bifurcation; 4. 0.66 mm distance between the anterior and posterior cerebral arteries; 5. left posterior cerebral artery; 6. rotated basilar artery bifurcation; 7. dorsum sellae; 8. A1 segment of the right anterior cerebral artery; 9. right optic canal.

**Figure 5:**
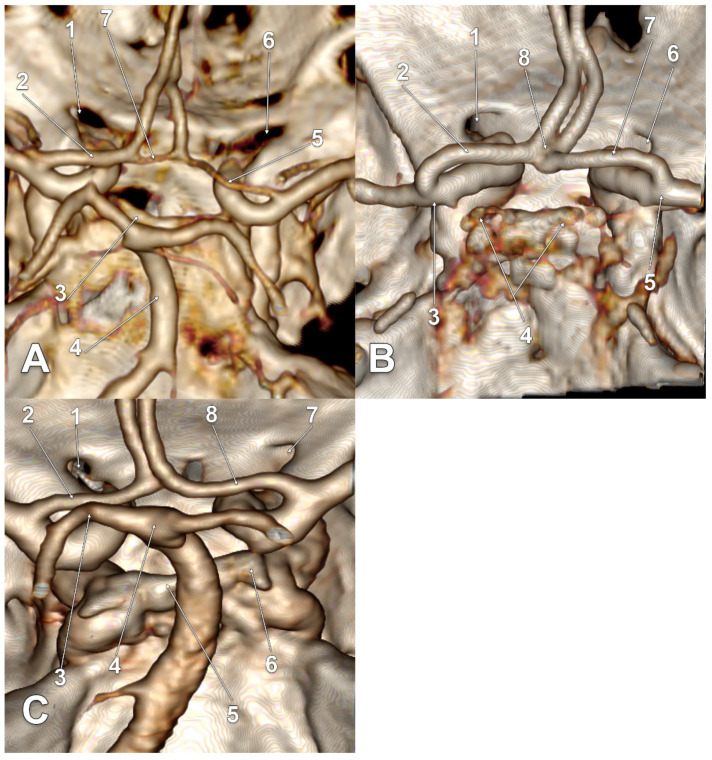
(**A**) Three-dimensional volume rendering of the skull base, posterosuperior view. Supracarotid infraoptic anterior cerebral arteries. Rotated anterior communicating artery. 1. Left optic canal; 2. A1 segment of the left anterior cerebral artery; 3. dorsum sellae; 4. basilar artery; 5. hypoplastic A1 segment of the right anterior cerebral artery; 6. right optic canal; 7. rotated anterior communicating artery. (**B**) Three-dimensional volume rendering of the skull base, posterosuperior view. Supracarotid infraoptic anterior cerebral arteries. 1. Left optic canal; 2. A1 segment of the left anterior cerebral artery; 3. left internal carotid artery bifurcation; 4. posterior clinoid processes; 5. right internal carotid artery bifurcation; 6. right optic canal; 7. A1 segment of the right anterior cerebral artery; 8. fenestrated anterior communicating artery. (**C**) Three-dimensional volume rendering of the skull base, posterosuperior view. Supracarotid infraoptic anterior cerebral arteries. Rotated basilar artery bifurcation. 1. Left optic canal; 2. A1 segment of the left internal carotid artery; 3. left posterior cerebral artery, angled at 0.16 cm posterior to the ipsilateral A1 segment; 4. basilar artery bifurcation at 1.06 cm above the dorsum sellae; 5. dorsum sellae; 6. right posterior clinoid process; 7. right optic canal; 8. A1 segment of the right anterior cerebral artery.

In 21/145 cases (14.48%), infraoptic courses of the A1 segments of the ACAs were found for type two and type three. The main findings are presented in Figure 2, Figure 3, Figure 4 and Figure 5, and are resumed in Table 1. These variant arteries had horizontal medial courses beneath the optic nerves. Infraoptic A1 segments were found in 12 male and 9 female cases. Type two was found bilaterally in 16/145 cases (11.03%) for 9 males and 7 females. Unilateral evidence of type two was gathered in 3/145 cases (2.06%), 1 male (Figure 2A) and 2 female cases (Figure 2B,C). Typethree3 was found bilaterally in 2/145 cases (1.38%), both male (Figure 2A,D).

All infraoptic or infrachiasmatic A1 segments were found to originate from long intradural ICAs with horizontal courses at the level, or just slightly above the level, of the clinoid processes that determined the low bifurcations of those ICAs. Except one unilateral type two female case (Figure 2C), all the other ICAs giving off infraoptic A1 segments coursed posteriorly, instead of posterosuperiorly, to bifurcate at the level, or even posterior to the posterior clinoid processes (low bifurcations) (Figure 2D, Figure 3A–D, Figure 4A–D and Figure 5A–C). In the respective unilateral type two female case (Figure 2C), the ICAs had high bifurcations. On the left side, the ICA bifurcation was directed superiorly, and the A1 segment of the ACA looped above the optic nerve. The right ICA gave of a fetal type of PCA and then it curved anteriorly above the anterior clinoid process; its bifurcation was directed anteriorly, and the A1 segment of that ACA coursed above the anterior clinoid process to continue between the optic nerve and the C6 ICA (supraclinoid, infraoptic and supracarotid course).

In one bilateral type three male case (Figure 3A), as well as in one bilateral type two female case (Figure 5A), bilateral supracarotid courses of those A1 segments were found. In one unilateral type two male case (Figure 2A), as well in one bilateral type two male case (Figure 4C), the respective left A1 segments had equally supracarotid and infraoptic courses. In the unilateral type two female case previously described, the supraclinoid, infraoptic and supracarotid A1 segment was on the right side (Figure 2C).

In a unilateral type two male case, the left infraoptic A1 segment was hypoplastic (Figure 2A). In two bilateral type two female cases, the respective right A1 segment was also hypoplastic (Figure 5A).

In two bilateral type two female cases, the BA bifurcation was rotated, thus, the PCA almost contacted the A1 segment of the respective ACA (Figure 4D and Figure 5C).

## 4. Discussion

The infraoptic course of the A1 segment of the ACA is an extremely rare anatomic variant that was first encountered by Robinson in 1959 during an anatomic dissection [11,12,13]. In that case, the right ICA divided into the ACA and MCA immediately after piercing the dura mater, while the ophthalmic artery was replaced by an anastomosis of the middle meningeal and lacrimal arteries [12]. Therefore, Robinson’s finding was an early, low bifurcation of the intradural ICA. Here, we found a different variant of the infraoptic A1 segments leaving elongated intradural ICAs distally (late bifurcations of the ICAs). Therefore, Robinson’s variant could be distinguished as a “proximal infraoptic A1 ACA”, while ours could be regarded as a “distal infraoptic A1 ACA”. Seemingly, when the ICA bifurcated posteriorly at the level of the posterior clinoid processes, an infraoptic course of the A1 invariably occurs, as the ascending angle of the optic nerve of an average of 45^o^ would make it superiorly cross the ACA.

The proximal infraoptic A1 segment of the ACA left the intradural ICA near the level of the ophthalmic artery [14]. The ICA bifurcation in such cases is usually located at the proximal dural ring, at, above or below the level of origin of the ophthalmic artery [7]. Such proximal infraoptic A1 segments were rarely found leaving the extradural ICA [14]. Bilateral proximal infraoptic A1 segments of the ACAs were found arising inferior to the optic strut [7]. Few cases of bilateral proximal infraoptic A1 segments of the ACAs were reported until recently [4,5,7,13,15,16,17,18,19].

An infraoptic A1 segment could also be associated with fused pericallosal arteries, an anomalous origin of the ipsilateral ophthalmic artery from the external circulation and moyamoya disease, as well as with symptoms of optic nerve, chiasm compression or cerebral arteriovenous malformations [6,19].

Different authors reported cases of aneurysms associated with infraoptic ACAs [20]. These were found on the AComA or on the A1 or A2 segments of the ACA [4,5,7,8,13,18,20,21,22,23,24,25,26]. An aneurysm on an infraoptic A1 segment of an azygos ACA was also reported [14]. Approximately 44% of patients with an infraoptic A1 are associated with AComA aneurysms [7,13]. It is, therefore, important to recognize such anatomic variants for preoperative planning, especially for endovascular approaches [7].

**Figure 6:**
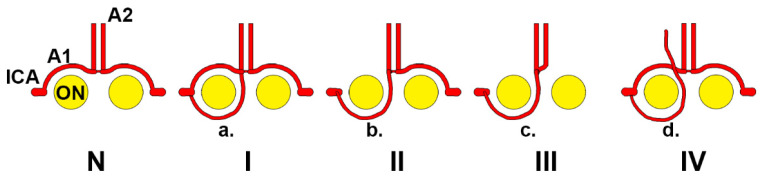
Wong’s types of anterior cerebral artery (ACA) in relation to the optic nerve. N: normal anatomy, the A1 (precommunicating) segment leaves the internal carotid artery (ICA) and has a supraoptic course to further continue with the A2 (postcommunicating) segment. Type I: The A1 segment has a supraoptic course, but an additional collateral (**a**) has an infraoptic course—it could be regarded as an ICA–ACA anastomosis. Type II: single, infraoptic A1 segment (**b**). Type III: in the absence of a contralateral A1 segment, the infraoptic artery becomes an infraoptic azygos ACA (**c**). Type IV: an infraoptic accessory ACA (**d**) is added to a normal ACA. Modified after [19].

Wong et al. (2008) documented the types of vascular configurations with proximal infraoptic A1 [19] (Figure 6). Types I and II can occur bilaterally [19]. The distal infraoptic A1 we demonstrated here corresponded to Wong’s type II.

Different authors reported “carotid–anterior cerebral artery” anastomoses with infraoptic courses [27,28,29,30,31,32,33] instead of infraoptic A1 ACAs. Such ICA–ACA anastomoses were found in 3/3,491 consecutive magnetic resonance angiographies, thus, in 0.086% [30]. Uchino (2017) distinguished a proximal infraoptic A1 segment from an ICA–ACA anastomosis: in cases with an ipsilateral A1 segment, the additional artery with an infraoptic course should preferably be regarded as an ICA–ACA anastomosis [34]. When unique, an infraoptic A1 segment is, anatomically, just a misplaced ACA. Yi et al. (2016) discussed the use of the terms “carotid–ACA anastomosis” (ICA–ACA anastomosis) vs. “infraoptic course of ACA” [33]. They considered that, based on the literature and embryology, the term “carotid–ACA anastomosis” should be preferred rather than “infraoptic ACA”, but as a morphologic and positional, and, thus, anatomical, description, the term “infraoptic ACA” has value [33].

Turnbull reported in 1962 a case with agenesis of the left ICA and a right ICA bifurcated after perforating the dura mater into the MCA, and an azygos ACA that coursed initially beneath the optic chiasm [35]. Therefore, Turnbull provided evidence of a proximal bifurcation of the ICA. Bosma later quoted the finding of Turnbull, but he described that Turnbull’s variant of the ACA “passed beneath the optic nerve” [36]. Therefore, an infrachiasmatic course of an A1 segment of ACA, different from an infraoptic one, was overlooked. In the present study, we distinguished the infraoptic course of the A1 segment (type two) from an infrachiasmatic one (type three). This distinction is highly relevant during microneurosurgical procedures approaching the optic nerve and chiasm.

Bosma (1977) reported the case of a patient with bilateral visual field disturbances in whom the optic chiasm exploration revealed a very low ICA bifurcation and an ipsilateral infraoptic right azygos ACA [36]. Bosma documented a previous report of Isherwood and Dutton (1969) of two cases in which the optic nerves were kinked by infraoptic ACAs [16,36].

Progressive visual loss commonly occurs in pituitary adenomas and associated lesions of the sella turcica [37]. The infraoptic A1 segment of the ACA, or an ICA–ACA anastomosis, could anatomically determine visual alterations, but, to our knowledge, it was commonly overlooked in previous studies as a potential etiology of such visual alterations.

The bilateral gross ectasia of ICAs extending into the MCAs was demonstrated in a patient with open-angle glaucoma; the authors discussed that neural imaging investigations should be considered if the presentation is not typical of a chronic bilateral optic neuropathy [38]. The authors’ three-dimensional volume rendering proof demonstrates supracarotid and infraoptic courses of the A1 segments of the ACAs, such as in the present study. The infraoptic or infrachiasmatic courses of the A1 segments of those ACAs were overlooked, although the authors explicitly described that the ICAs “encroached on the under surface of the optic chiasm” [38].

Kim et al. (2016) reported several cases of infraoptic A1 ACAs, but overlooked that, in one of those cases, the A1 segment of the ACA looped above the ICA before continuing with an infraoptic course [23]. This is a supracarotid course of an infraoptic A1 segment, such as the one we also found in the present study.

Infraoptic A1 segments of the ACA modify the neurosurgical triangles of the skull base. The opticocarotid triangle of Yaşargil is limited medially by the optic apparatus, laterally by the ICA and posteriorly by the A1 segment of the ACA [39]. A supracarotid infraoptic A1 segment would not modify the borders of this triangle, but the extremities of its posterior border would be repositioned: the lateral one—above the ICA—and the medial one—beneath the optic nerve. The clinoidal triangle of Dolenc corresponds to the interval between the optic nerve and the anterior clinoid process [39]. A supraclinoid and supracarotid course of the A1 segment of the ACA, such as we found in this study, would unexpectedly appear within the clinoidal triangle of Dolenc. Moreover, during an anterior clinoidectomy, an unexpected supraclinoid A1 segment of the ACA could be accidentally damaged. The ventral thalamopeduncular junction, the anteroinferior basal ganglia and the ICA bifurcation could be accessed via the supracarotid triangle [39,40]. The supracarotid triangle is a flat triangle in the supracarotid space, between the initial segment of the A1 ACA, medially, and of the M1 segment of the MCA; the upper base of this triangle is the basomedial frontal lobe (the medial orbital gyrus, lateral to the gyrus rectus and olfactory tract) overlying those arterial segments [41]. Posterior to the medial orbital gyrus are the anterior olfactory stria and the anterior perforated substance that comprises the inferior aspect of the basal ganglia [41]. A dissection of this supracarotid triangle involves opening the space around the A1 segment of the ACA until the A1 and M1 segments have been completely mobilized and the bifurcation of the supraclinoid ICA is observed [40]. Surgeons should be aware that, in cases with an elongated ICA and a supracarotid course of a distal infraoptic A1 segment of the ACA, the supracarotid triangle is likely to be displaced posteriorly, and the inferior medial limit would be represented by the A1 segment coursing over the ICA and continuing medially beneath the optic nerve.

For a vascular or skull base neurosurgeon understanding the anatomy of the skull base is crucial. This is especially true in the anterior cranial fossa, because a wide variety of vascular and tumoral pathology develops in this region. This is a strong reason for knowing the vascular anatomy and accurately identifying anatomical variants preoperatively. Unexpected vascular variants can pose intraoperative problems, even for experienced neurosurgeons. In the middle region of the skull base, at the level of the sphenoid body and lesser wings, thus, anatomically related to the anterior circle of Willis, significant tumoral pathology may be present: (a) meningiomas of the sphenoid small wing, anterior clinoid processes or sellar tubercle; (b) pituitary adenomas; and (c) craniopharyngiomas. The preoperative identification of various tumors that may interfere with an elongated ICA and infraoptic A1 is important for the successful removal of such tumors. Arterial variants have unusual trajectories that may interfere with neurosurgical corridors. The tumors appear first in the neurosurgical field and deform or obscure normal anatomical elements, such as the optic nerve, ICA and its branches. Endoscopic transsphenoidal surgery for pituitary adenoma can also lead to vascular damage during a dissection or removal of the tumor capsule.

## 5. Conclusions

An infraoptic course of the A1 segment of the ACA could leave elongated ICAs. Such distal infraoptic ACAs could have a supracarotid course, above the ICA, or even a supraclinoid one, above the anterior clinoid process. Therefore, common neurosurgical landmarks are modified.

## Figures and Tables

**Table 1:** Anatomical variation in cases with infraoptic courses of the A1 segment (IO A1) of the anterior cerebral artery (ACA). SCar: supracarotid course of the IO A1. SClin: supraclinoid course of the IO A1. AComA: anterior communicating artery. PCA: posterior cerebral artery. M: male. F: female.

Gender	Right Side	Left Side	Other Variations	Figure
M	type 3 IO A1 SCar	type 3 IO A1 SCar	high BA bifurcation	Figure 3A
M	type 3 IO A1	type 3 IO A1		Figure 2D
M	type 2 IO A1	type 1 IO A1 SCar	fenestrated AComA, hypoplastic left A1	Figure 2A
M	type 2 IO A1	type 2 IO A1 SCar		Figure 4C
M	type 2 IO A1	type 2 IO A1		Figure 4A
M	type 2 IO A1	type 2 IO A1		–
M	type 2 IO A1	type 2 IO A1		–
M	type 2 IO A1	type 2 IO A1		–
M	type 2 IO A1	type 2 IO A1		Figure 5B
M	type 2 IO A1	type 2 IO A1		–
M	type 2 IO A1	type 2 IO A1		–
M	type 2 IO A1	type 2 IO A1		–
F	type 2 IO A1	type 2 IO A1	rotated BA, left PCA displaced towards the ipsilateral A1 ACA	Figure 4D
F	type 2 IO A1	type 2 IO A1	duplicated AComA	Figure 4B
F	type 2 IO A1 SCar SClin	type 1 IO A1	supraclinoid right A1	Figure 2C
F	type 2 IO A1	type 2 IO A1		Figure 3D
F	type 2 IO A1	type 2 IO A1		Figure 3B,C
F	type 2 IO A1	type 2 IO A1	hypoplastic right A1	
F	type 2 IO A1 SCar	type 2 IO A1 SCar	rotated AComA, hypoplastic right A1	Figure 5A
F	type 2 IO A1	type 2 IO A1	rotated BA, left PCA displaced towards the ipsilateral A1 ACA	Figure 5C
F	type 1 IO A1	type 2 IO A1	left bihemispheric ACA	Figure 2B
